# Progress of targeted FOX family therapy in ovarian cancer

**DOI:** 10.3389/fphar.2025.1604998

**Published:** 2025-07-17

**Authors:** Hairong Zhang, Cuiping Gong, Xin Lv

**Affiliations:** Department of Obstetrics and Gynecology, Shandong Provincial Third Hospital, Shandong University, Ji’nan, China

**Keywords:** ovarian cancer, FOX family, targeted therapy, FOXA, FOXC, FOXM, FOXO, FOXP

## Abstract

Ovarian cancer (OC) remains one of the most lethal malignancies affecting women, largely due to its asymptomatic onset and the consequent challenges in early detection and diagnosis. This often results in delayed treatment and poor clinical outcomes. Among gynecological cancers, OC exhibits the highest mortality rate. While current therapeutic approaches such as surgery and chemotherapy provide initial clinical benefit, they are frequently undermined by high rates of recurrence and metastasis. Moreover, the pronounced heterogeneity of OC further complicates treatment, highlighting the urgent need for novel therapeutic targets and more effective strategies. The forkhead box (FOX) family of transcription factors comprises a large group of proteins involved in regulating gene expression across various biological processes. Dysregulation of FOX family members has been implicated in aberrant cellular behaviors, including uncontrolled proliferation, resistance to apoptosis, enhanced invasiveness, metastatic potential, and the development of drug resistance. Importantly, the functional roles of individual FOX proteins vary significantly depending on the tumor context, reflecting the functional diversity of this family. This review aims to provide a comprehensive overview of the emerging roles of FOX family members in the pathogenesis and progression of OC, as well as recent advances in FOX-targeted therapeutic strategies.

## Introduction

Ovarian cancer (OC), a primary malignancy of the female reproductive system, ranks among the most aggressive and fatal gynecologic cancers worldwide. Its clinical course is marked by rapid progression, high relapse rates, and limited survival outcomes, contributing to a notably high mortality rate (Stewart et al., 2019). The deep anatomical location of the ovaries within the pelvic cavity often results in vague or asymptomatic early-stage disease, and currently, there are no reliable or widely available screening tools for early detection. Consequently, most patients are diagnosed at an advanced stage, when therapeutic options are less effective and the 5-year survival rate drops to approximately 30%–40%. The pathogenesis of OC is multifactorial, involving a complex interplay of genetic mutations, hormonal influences, and environmental exposures. Compounding its clinical challenges, OC is highly invasive and prone to early metastasis, frequently spreading throughout the peritoneal cavity and to distant sites, thereby complicating disease control and treatment strategies.

The forkhead box (FOX) family of transcription factors represents an evolutionarily conserved group of proteins that play essential roles in regulating a wide range of cellular processes (Laissue, 2019). This family consists of numerous members classified into 19 subfamilies—such as FOXA, FOXB, and FOXC—based on similarities in structure and function. FOX proteins are central to many physiological processes, including embryogenesis, cellular differentiation, metabolism, and immune regulation, and are increasingly recognized for their involvement in cancer development and progression (Golson and Kaestner, 2016). Notably, FOX family members exhibit diverse and sometimes opposing functions: while the FOXO subfamily is generally associated with tumor suppression and regulation of apoptosis, FOXM1 has been implicated in promoting cell cycle progression and oncogenic transformation. Each FOX protein contains a conserved DNA-binding domain that enables it to recognize specific DNA sequences, functioning as a molecular switch that controls the expression of downstream genes. Through this mechanism, FOX transcription factors exert precise control over cellular behavior and fate decisions, with dysregulation often contributing to oncogenesis.

## FOXA1

The FOXA family includes FOXA1 (also known as hepatocyte nuclear factor 3A [HNF3A]); FOXA2 (also known as HNF3B) and FOXA3 (also known as HNF3G) (Bernardo and Keri, 2012). FOXA1 was first isolated from the liver (Bernardo and Keri, 2012). FOXA1 is located on chromosome 14q21.1. FOXA1 is expressed in various organ tissues, including the pancreas, breast, prostate, liver, lungs, brain, gastrointestinal tract, and kidneys (Golson and Kaestner, 2016). The FOXA expression is relatively low in normal ovarian tissues, but significantly increased in ovarian cancer tissues (Wang K. et al., 2018; Sheta et al., 2021; Zheng et al., 2020), and high FOXA expression is positively correlated with poor prognosis (Wang K. et al., 2018). However, it should be emphasized that the high FOXA1 expression is most evident in mucinous ovarian cancer (Sheta et al., 2021; Karpathiou et al., 2017). In addition, this study demonstrated that in normal structures, ciliated fallopian tube epithelial cells, Walthard nests, and transitional metaplasia within the mesothelial lining of the fallopian tube express high levels of FOXA1 (Karpathiou et al., 2017). In addition to its role as a classical transcription factor, FOXA1 protein functions as a pioneer factor by interacting closely with chromatin so as to promote the binding of other transcription regulator, such as estrogen receptor (ER) and androgen receptor (AR). Therefore, in breast cancer and prostate cancer, the high expression of ER and AR is often accompanied by the high expression of FOXA1 (Wang K. et al., 2018; Lupien et al., 2008; Wang et al., 2009). Although the ER expression is extremely low in normal ovarian tissues, it is significantly increased in ovarian cancer. Unfortunately, there is currently insufficient evidence to confirm the correlation between FOXA1 and ER expressions in ovarian cancer and it is unclear whether the high FOXA1 expression and poor prognosis in ovarian cancer are influenced by ER expression.

Currently, the regulation of the FOXA1 expression is affected by diverse mechanisms such as acetylation (Lou et al., 2022) and miRNA (Zheng et al., 2020) in ovarian cancer. Therefore, targeting HDAC3 and LncRNA SNHG17 are the potential targets for intervention in the FOXA1 expression. In addition, the loss of expression of the transcription factor ID4 can increase the FOXA expression, albeit the specific underlying mechanism remains unclear (Table 1).

**TABLE 1:** Drug therapy targeting FOX family members in ovarian cancer.

Drug	Target	Target effect	Biological effects	References
DFOG	FOXM1	Downregulation	Inhibit the activity of stem cells	Ning et al. (2014)
ATRA	FOXM1	Downregulation	Inhibit the activity of stem cells	Young et al. (2015)
JQ1	FOXM1	Downregulation	Pro-apoptosis	Zhang et al. (2016), Chen et al. (2025)
domatinostat	FOXM1	HDAC inhibition	anti-proliferation	Nakagawa-Saito et al. (2023)
FDI-6	FOXM1	Suppress transcriptional activity	Inhibit cell activity	Lee et al. (2021)
Myricetin	FOXM1	Ubiquitination degradation by downregulated CD147	Inhibit cell vitality	Chen L. et al. (2024)
NB-73 、NB-115	FOXM1	Ubiquitination degradation	Pro-apoptosis	Liu et al. (2024)
thiostrepton	FOXM1	Downregulation	Anti-resistance	Westhoff et al. (2017)
apigenin	FOXO3	Phosphorylation by AKT	Oxidative stress and cell apoptosis	Castrillon et al. (2003)
selinexor (KPT-330)	FOXO1	Nuclear localization by inhibition of XPO-1	Inhibit cell vitality	Corno et al. (2018)
Casticin	FOXO3	Upregulation	pro-apoptosis	Jiang et al. (2013)
10,058-F4	FOXO	Upregulation	Anti-proliferation, pro-apoptosis, pro- autophagic cell death	Ghaffarnia et al. (2021)
Melatonin	FOXO3a	Nuclear localization	Prevention of cisplatin-induced loss of primordial follicles	Jang et al. (2016)
Quercetin	FOXO3a	Nuclear localization by Inhibition of PTEN	Reduced apoptosis of the growing follicles	Li M. et al. (2021)
niraparib	FOXQ	Ubiquitination degradation		Wu et al. (2024)

**FIGURE 1:**
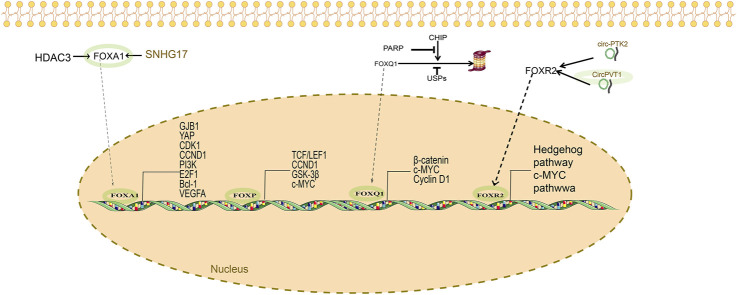
FOXA/FOXP/FOXQ1/FOXR2 and ovarian cancer.

Pats studies have demonstrated that FOXA1, as a transcription factor, affects ovarian cancer prognosis by upregulating gap junction protein β-1 (GJB1) (Yang et al., 2021). The underlying mechanism involves the “ECM receptor interaction” and “focal adhesion” pathways. GJB1, a downstream signal of FOXA1, is a potential therapeutic target. Wang et al. (Wang et al., 2017) reported that, in FOXA1-silenced ovarian cancer cell lines, cell proliferation, migration, and invasion are reduced. The apoptotic activity is upregulated with the induction of the s-phase blockade. Silencing of FOXA1 protein can reduce the expression of several factors, including YAP, CDK1, CCND1, PI3K, E2F1, Bcl-2, and VEGFA proteins. Past studies have also demonstrated that FOXA1 can interact with the connective tissue growth factor (CTGF) promoter, thereby influencing the development of drug resistance in ovarian cancer (Wang et al., 2022) (Figure 1).

## FOXC

The FOXC subfamily comprises FOXC1 and FOXC2, which are predominantly expressed in the cardiovascular system (Tan et al., 2024), lymphatic vasculature (Kurup et al., 2023), and ocular tissues (Seifi and Walter, 2018). However, these transcription factors are also present in other organs, including the reproductive, respiratory, and digestive systems. Elevated levels of FOXC1 and FOXC2 have been reported in various cancers, such as breast cancer, hepatocellular carcinoma, and lymphoma (Wang T. et al., 2018; Elian et al., 2018). These factors contribute to tumor progression by promoting cell proliferation, metastasis, epithelial–mesenchymal transition (EMT), angiogenesis, and lymphangiogenesis (Hollier et al., 2013).

**FIGURE 2:**
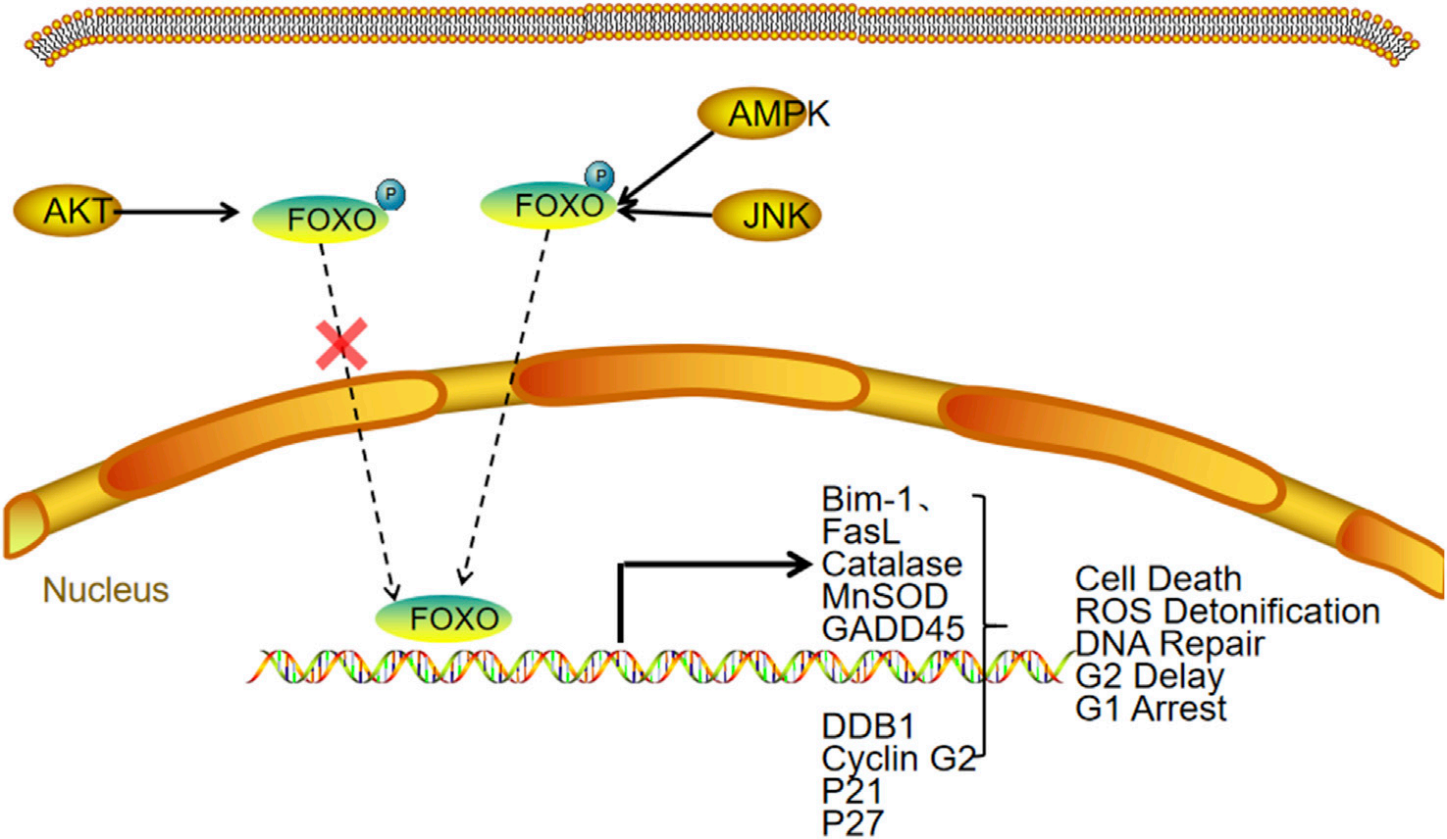
FOXC and ovarian cancer.

Wu et al. demonstrated that high FOXC1 expression may facilitate EMT in OC cells, thereby accelerating tumor invasion and metastasis (Wang L. Y. et al., 2016). This upregulation of FOXC1 might be linked to increased levels of chromodomain helicase DNA binding protein 1-like (CHD1L) in OC. Notably, CHD1L overexpression enhances the production of circ-PTK2, which acts as a molecular sponge for miR-639, ultimately resulting in elevated FOXC1 expression. Lin et al. reported that FOXC2 overexpression induces the expression of stanniocalcin, which directly binds to integrin β6, activating the PI3K signaling pathway. This activation upregulates lipid metabolism-related genes, including UCP1, TOM20, and perilipin 1, thereby promoting lipid metabolic processes. Furthermore, their study identified the FOXC2/STC1/ITGB6 signaling axis as a contributor to cisplatin resistance *in vitro* (LIN et al., 2022) (Figure 2).

Interestingly, high FOXC1 expression may serve as a marker for benign ovarian tumors and correlate with a favorable prognosis in OC (Wang L. Y. et al., 2016). This discrepancy indicates that the precise role of FOXC proteins in OC remains to be fully elucidated.

## FOXM1

FOXM1 is typically highly expressed in organs with rapid cellular turnover, such as the small intestine, colon, and thyroid gland, but shows relatively low expression in normal ovarian tissue (Wierstra and Alves, 2007). However, FOXM1 expression is significantly upregulated in a wide range of cancers, including OC (Golson and Kaestner, 2016; Wen et al., 2014). Data from The Cancer Genome Atlas reveal that the FOXM1 gene locus is amplified in approximately 12% of high-grade serous ovarian carcinomas, a higher frequency than in any other tumor type. This amplification correlates with increased FOXM1 expression and poorer patient survival (Zhang et al., 2023). Among the three known FOXM1 isoforms, FOXM1c is predominantly expressed in epithelial OC. Moreover, the combined deletion of tumor suppressors Rb1 and Tp53 synergistically induces FOXM1 expression in mouse ovarian surface epithelial cells (Barger et al., 2015). FOXM1 overexpression is observed not only in serous OC (Zhang et al., 2023; Barger et al., 2021) but also in non-serous subtypes such as clear cell carcinoma and endometrioid carcinoma (Tassi et al., 2017).

High FOXM1 expression drives OC cell proliferation (Li et al., 2020), invasion (Li et al., 2020), metastasis (Pratheeshkumar et al., 2018; Zhang Z. et al., 2020; Parashar et al., 2020), and chemotherapy resistance (Zhou Z. Y. et al., 2022; Nakagawa-Saito et al., 2023). Additionally, FOXM1 supports the maintenance of OC stem cell properties (Ning et al., 2014; Young et al., 2015; Sher et al., 2022) and contributes to metabolic reprogramming within the tumor microenvironment (Wang Y. et al., 2016). Its downstream targets include keratins KRT5 and KRT7 (Zhang Z. et al., 2020); glucose transporters GLUT1 and hexokinase 2 involved in glycolysis (Wang Y. et al., 2016); β-catenin, a key regulator of cell adhesion and Wnt signaling (Pratheeshkumar et al., 2018; Chiu et al., 2015); survivin, which inhibits apoptosis (Nakagawa-Saito et al., 2023); and cell cycle regulators cyclin F and KIF20A (Li et al., 2020), as well as CDCA5 (Zhang et al., 2023). FOXM1 also regulates stemness markers such as CD133, CD44, and ALDH1 (Ning et al., 2014).

### Regulation of FOXM1 expression in OC

Upstream transcription factor regulation: The transcription factor ETV5 is markedly overexpressed in OC and functions as a key activator of FOXM1 transcription. By enhancing FOXM1 expression, ETV5 promotes the upregulation of cell adhesion molecules critical for tumor cell attachment, invasion, and peritoneal dissemination (Llauradó et al., 2012). Notably, FOXO3 also acts as a transcriptional activator of FOXM1, adding another layer to FOXM1 gene regulation (Jiang et al., 2013).

Gene amplification and chromosome remodeling: According to The Cancer Genome Atlas data, approximately 12% of high-grade OCs exhibit amplification of the FOXM1 locus. Additionally, epigenetic modifications such as histone deacetylation can alter chromatin structure to increase FOXM1 transcriptional activity, facilitating a more open chromatin conformation conducive to gene expression.

Post-transcriptional regulation: Several non-coding RNAs regulate FOXM1 expression by interacting with its mRNA, primarily targeting 3′UTR. Circular RNAs such as circ_0025033 promote FOXM1 upregulation in paclitaxel (PTX)-resistant OC cells by sequestering miR-532-3p, thereby relieving its inhibitory effect on FOXM1 and enhancing malignancy (Huang et al., 2022). Similarly, circPVT1 binds miR-149-5p to increase FOXM1 expression, influencing OC cell viability and migration (Li M. et al., 2021). Long non-coding RNAs like lncRNA PVT1 act as “molecular sponges” by binding miR-370, leading to the derepression of FOXM1 and subsequent promotion of cell proliferation, migration, and invasion (Yi et al., 2020). Furthermore, microRNAs including miR-532-3p, miR-149-5p, miR-370, and miR-877 directly target the 3′UTR of FOXM1 mRNA, inhibiting translation or inducing degradation. For instance, miR-877 overexpression suppresses OC cell migration and invasion by downregulating FOXM1 (Fang et al., 2021).

**FIGURE 3:**
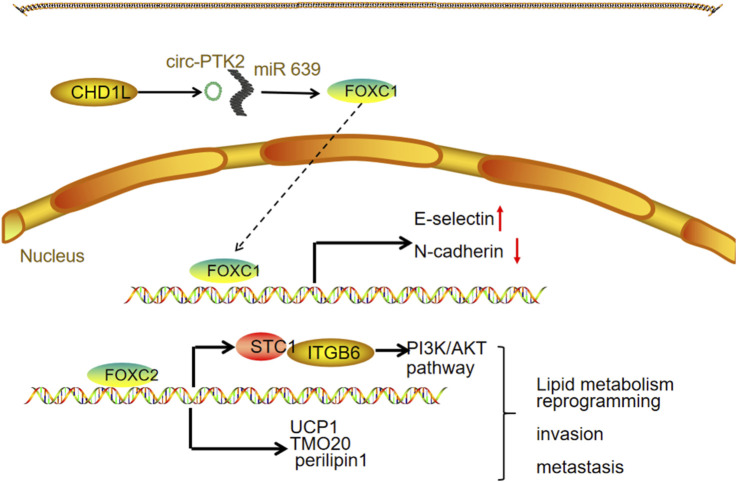
FOXM1 and ovarian cancer.

Post-translational modifications: FOXM1 protein function is tightly controlled by various PTMs, such as phosphorylation, ubiquitination, SUMOylation, and acetylation. Phosphorylation is a pivotal mechanism regulating FOXM1’s nuclear localization and transcriptional activity. Kinases involved include those in the PI3K/AKT/mTOR, GSK-3β, and ERK pathways, which phosphorylate FOXM1 to enhance its nuclear import and activation. Additionally, ubiquitination, SUMOylation, and acetylation modulate FOXM1 stability and activity, thereby fine-tuning its oncogenic functions (Figure 3).

Current strategies to target FOXM1 in OC encompass multiple approaches, including modulation of FOXM1 gene expression, interference with post-transcriptional mechanisms, and direct inhibition of FOXM1 protein function to block its binding to downstream promoters.

The transcription factor ETV5 is significantly upregulated in ovarian tumor samples and transcriptionally activates FOXM1. Downregulation of ETV5 reduces FOXM1 expression, highlighting a direct regulatory relationship. Moreover, increased ETV5 expression correlates with elevated FOXM1 transcript levels in ovarian tumor samples. ETV5 also modulates the expression of cell adhesion molecules and improves the survival of OC cells under anchorage-independent conditions, suggesting a key role in promoting tumor cell dissemination and peritoneal metastasis (Llauradó et al., 2012).

Casticin, a natural multi-methoxy flavonoid, has demonstrated anticancer activity in OC. Its pro-apoptotic effects are likely mediated through activation of FOXO3a, which in turn inhibits FOXM1 expression, thereby reducing cancer cell proliferation and survival (Jiang et al., 2013).

CD147 contributes to cisplatin resistance in OC via a unique mechanism involving proteasome-mediated degradation of FOXM1. This regulation is closely linked to DNA damage repair processes in OC cells. Licartin, an antibody drug approved by the China National Medical Products Administration, targets CD147 and may have therapeutic potential in enhancing cisplatin sensitivity in OC (Wang M. et al., 2024). Additionally, myricetin, a natural CD147 inhibitor, increases cisplatin sensitivity by indirectly promoting FOXM1 degradation (Chen L. et al., 2024).

NB compounds exhibit selective inhibitory activity against FOXM1, with minimal off-target effects on other FOX family members. These compounds promote proteasome-mediated degradation of the FOXM1 protein, resulting in decreased expression at both the mRNA and protein levels and suppressing the transcription of FOXM1-regulated oncogenic targets (Liu et al., 2024).

Bromodomain and extraterminal (BET) proteins, which act as epigenetic readers by recognizing acetylated lysine residues on histones, regulate the transcription of oncogenes including FOXM1. Inhibition of BET proteins suppresses the proliferation and metastatic capacity of OC cells. Importantly, BET inhibitors can restore drug sensitivity in resistant OC cells, including cisplatin and PARP inhibitors (Andrikopoulou et al., 2021).

Domatinostat, a selective class I HDAC inhibitor currently in clinical development, has shown promising activity in OC. Moreover, domatinostat reduces both protein and mRNA levels of FOXM1 and survivin, thereby impairing cell viability (Nakagawa-Saito et al., 2023) (Table 1).

Both circ_0025033 and FOXM1 are highly expressed in OC tissues and cell lines, while miR-532-3p is significantly downregulated, especially in PTX-resistant OC cells. Knockdown of circ_0025033 leads to reduced PTX resistance, diminished migration and invasion capacity, and enhanced apoptosis in PTX-resistant cells. Mechanistically, circ_0025033 functions as a competing endogenous RNA by sponging miR-532-3p, thereby upregulating FOXM1 expression. Silencing circ_0025033 relieves this repression, increasing miR-532-3p availability and consequently downregulating FOXM1, ultimately inhibiting the malignant phenotype of PTX-resistant OC cells (Huang et al., 2022) (Table 2).

**TABLE 2:** Effect of RNA interference on Fox members in ovarian cancer.

Type	Target	Target effect	Biological effects	References
LncRNA SNHG17	FOXA1	Upregulation	proliferation, migration and invasion	Zheng et al. (2020)
Circ_0025033	FOXM1	Upregulation	PTX Chemosensitivity	Huang et al. (2022)
Circular PVT1	FOXM1	Upregulation	Promote cell vitality and migration	Li J. et al. (2021)
LncRNA PVT1	FOXM1	Upregulation	proliferation, migration and invasion	Yi et al. (2020)
MiR-877	FOXM1	Downregulation	Inhibit cell migration and invasion	Fang et al. (2021)
circCELSR1	FOXR 2	Upregulation	PTX Chemosensitivity	Liang et al. (2023)

PVT1 is an ovarian-specific gene that is overexpressed in multiple cancers, including OC. High PVT1 expression is positively associated with poor prognosis in patients with OC. Circular PVT1 enhances FOXM1 expression by binding to miR-149-5p, thereby promoting the viability and migration of OC cells (Li M. et al., 2021).

PVT1 also acts as a competing endogenous RNA by sponging miR-370 through two binding sites. This interaction facilitates malignant behaviors such as cell proliferation, migration, and invasion. Notably, the introduction of miR-370 mimics reverses these oncogenic effects, further confirming the regulatory axis of PVT1 and miR-370 in OC (Yi et al., 2020). MiR-877 is expressed at low levels in OC tissues and cell lines. Its overexpression significantly inhibits cell migration and invasion. FOXM1 has been identified as a direct target of miR-877, and miR-877 exerts its tumor-suppressive function by downregulating FOXM1 expression in OC cells (Fang et al., 2021).

Lysophosphatidic acid (LPA) upregulates the expression of FOXM1 splicing variants in epithelial OC cell lines—OVCA433, CAOV3, and OVCAR5—in a time- and dose-dependent manner. This upregulation is mediated through the LPA1–3 receptors and involves both Gi–PI3K–AKT and G12/13–Rho–YAP signaling pathways. Silencing FOXM1 significantly impairs tumor formation and metastasis and downregulates FOXM1 target genes involved in proliferation, migration, and invasion, suggesting FOXM1 is a key effector in LPA-induced tumorigenicity and ascites formation (Fan et al., 2015).

DFOG suppresses the stem cell-like characteristics of OC stem-like cells by downregulating FOXM1 expression (Ning et al., 2014). Aldehyde dehydrogenase 1 (ALDH1) activity is positively correlated with OC cell stemness and regulates FOXM1 and Notch1 expression. All-trans retinoic acid inhibits ALDH1 expression, thereby impairing tumor formation, sphere formation, cell migration, and invasion. Thus, all-trans retinoic acid exerts antitumor effects by suppressing the ALDH1/FOXM1/Notch1 signaling pathway (Young et al., 2015).

The synthetic compound XST-20 targets the DNA-binding domain of FOXM1, effectively inhibiting its transcriptional activity. Treatment of A2780 and SKOV3 OC cells with XST-20 results in decreased cyclin D expression and increased levels of p21 and p27, leading to enhanced apoptosis and reduced cell proliferation (Zhang et al., 2022). Thiostrepton, another FOXM1-targeting agent, inhibits the expression of FOXM1 and its downstream effectors CCNB1 and CDC25B, ultimately inducing cancer cell death. However, the precise mechanisms underlying this inhibition remain to be elucidated (Westhoff et al., 2017).

## FOXO

The four subtypes of FOXO proteins—FOXO1, FOXO3, FOXO4, and FOXO6—share high sequence homology and are confirmed to be expressed in mammalian cells (Santos et al., 2023). As critical transcription factors, FOXO proteins translocate from the cytoplasm to the nucleus, where they regulate the transcription of numerous target genes. In addition to their role in transcriptional control, FOXO proteins also interact with other cellular proteins to modulate their function and activity (Liu et al., 2015).

FOXO3a is essential for ovarian follicle development (Meerschaut et al., 2017). Female mice lacking Foxo3a exhibit a distinctive ovarian phenotype characterized by widespread follicular activation, leading to premature oocyte depletion, early loss of follicular function, and secondary infertility. These findings establish FOXO3a as a key inhibitory regulator of early follicular activation during ovarian development (Castrillon et al., 2003). FOXO1 is expressed in granulosa cells during fetal, prepubertal, and adult stages in rhesus monkeys (Ting and Zelinski, 2017). In contrast, FOXO3 expression is sparsely distributed in germ cells undergoing active mitosis, and its expression markedly declines following follicle formation in fetal macaque ovaries. FOXO3 exhibits minimal inter-individual variability in prepubertal ovaries and is generally absent in adult ovaries; however, it remains detectable in specific follicular and stromal cells within both prepubertal and adult ovaries.

FOXO3a expression is absent in serous tubal intraepithelial carcinomas and high-grade serous carcinomas, whereas it is present in normal fallopian tube epithelium (Van Der Ploeg et al., 2022; Lu et al., 2012). A reduction in FOXO expression has also been linked to the development of drug resistance in OC (Shi et al., 2021).

**FIGURE 4:**
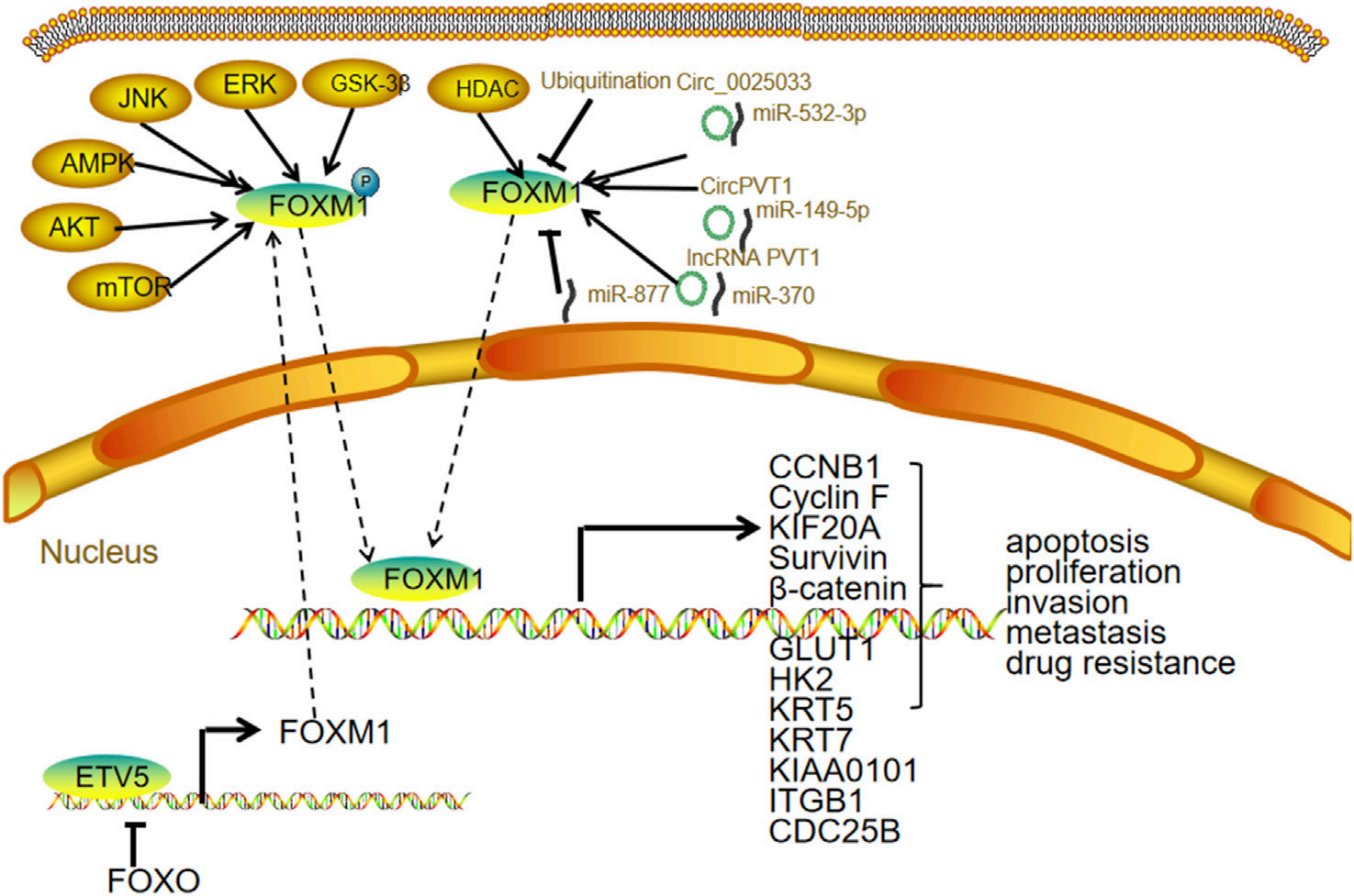
FOXO and ovarian cancer.

FOXO activity is primarily regulated through post-translational modifications, including phosphorylation, acetylation, ubiquitination, methylation, glycosylation, and nitrosation (Santos et al., 2023). Among these, phosphorylation plays a central role in modulating FOXO function, with outcomes that depend on the specific phosphorylation sites involved. The PI3K/AKT signaling pathway is a major regulator of FOXO phosphorylation, promoting its export from the nucleus to the cytoplasm and thereby decreasing its transcriptional activity (brown and Webb, 2018). Conversely, stress-activated kinases such as JNK, MST1, and AMPK phosphorylate FOXO in a way that enhances its nuclear retention and transcriptional activity. Phosphorylated FOXO proteins are also more prone to degradation via the ubiquitin–proteasome pathway, resulting in reduced protein levels (Zeng et al., 2025). FOXO expression can also be transcriptionally regulated. For instance, deprivation of growth factors leads to a reduction in the mRNA levels of FOXO1, FOXO3, and FOXO4 (Essaghir et al., 2009) (Figure 4).

Conversely, increased levels of reactive oxygen species, nutrient deprivation, and DNA damage can activate FOXO to restore cellular homeostasis (Wang Z. et al., 2016).

The following are the current strategies to therapeutically target FOXO: (1) modulating upstream signaling pathways, such as PI3K/AKT or AMPK, to influence FOXO activity; (2) acting directly on FOXO proteins; (3) disrupting interactions between FOXO and its binding partners; and (4) targeting FOXO downstream effectors.

4-Vinylcyclohexene diepoxide induces loss of follicular cells at all developmental stages, contributing to ovarian dysfunction—an effect potentially mediated by reactive oxygen species overproduction and oxidative stress. Natural compounds such as apigenin (Castrillon et al., 2003) and quercetin (Li J. et al., 2021), which are active components of traditional Chinese medicine, inhibit oxidative stress and apoptosis via the AKT/FOXO3a signaling pathway, thereby alleviating ovarian dysfunction.

Aulosirazoles B and C are compounds that activate FOXO, promoting the nuclear accumulation of FOXO3a in OVCAR3 cells (Davis et al., 2022). Exportin 1 (XPO1), also known as chromosome region maintenance 1, is a nuclear export protein responsible for transporting leucine-rich proteins from the nucleus to the cytoplasm. XPO1 is also involved in the nuclear export of FOXO. Studies have shown that the XPO1 inhibitor selinexor (KPT-330) significantly enhances FOXO nuclear localization in OC cells, inhibits cell proliferation, and increases tumor cell sensitivity to platinum-based chemotherapy (Corno et al., 2018). However, it is important to note that not all XPO1 inhibitors exert FOXO-dependent inhibitory effects in OC.

## FOXP

The FOXP family comprises four members: FOXP1, FOXP2, FOXP3, and FOXP4. These transcription factors play critical roles in regulating genes involved in immune responses, carcinogenesis, development, differentiation, and angiogenesis (Zhou Y. et al., 2022; Kim et al., 2019). FOXP1, FOXP2, and FOXP4 are highly expressed in the nervous system, where they contribute to the regulation of brain development and function (Co et al., 2020; Hickey et al., 2019). FOXP3, although structurally similar to other FOXP members, primarily functions in the development and maintenance of regulatory T cells (Tregs), thereby sustaining immunosuppressive activity (Chen S. et al., 2024). Notably, FOXP proteins exhibit dual roles in tumorigenesis, acting either as oncogenes or tumor suppressors depending on the cancer type, and their expression is variably associated with patient survival outcomes (Kamal et al., 2024; Akimova et al., 2024).

FOXP1 may exhibit functions similar to those of FOXA1, which, as previously discussed, is closely associated with steroid hormone receptors and may influence the progression of hormone-dependent tumors. In breast cancer, FOXP1 expression is positively correlated with estrogen receptor alpha (ERα) levels. Patients lacking both ERα and FOXP1 exhibit significantly shorter progression-free survival (Rayoo et al., 2009). Estrogen also induces FOXP1 expression (Shigekawa et al., 2011).

In normal ovarian tissue, ERα expression is generally absent or minimal, but it becomes markedly upregulated in OC. Higher ERα expression correlates with increased malignancy. Conversely, ERβ is highly expressed in normal ovarian tissue but significantly downregulated in OC. The relative overexpression of ERα compared to ERβ is considered a hallmark of OC. A study by Zhang et al. (Hu et al., 2015) reported a progressive increase in FOXP1 expression across normal ovarian tissue, benign tumors, borderline tumors, and malignant OCs, while ERα expression exhibited a decreasing trend. Interestingly, the expression pattern of ERβ closely mirrored that of FOXP1. Furthermore, the study observed a shift in FOXP1 localization from the nucleus to the cytoplasm with increasing tumor malignancy, suggesting reduced nuclear FOXP1 expression and increased cytoplasmic staining. This pattern opposes that of ERα and aligns with ERβ expression. The authors speculated that hypermethylation of the promoter regions of FOXP1 and ERβ contributes to their downregulation in OC. Estrogens appear to play a central role in modulating the expression and activity of FOXP1 and ERβ, exerting inhibitory effects on tumor proliferation and invasion. These findings suggest that targeting estrogen signaling pathways may offer novel therapeutic strategies for OC (Figure 1).

Bioinformatics analyses demonstrated that FOXP4 mRNA expression is significantly elevated in OC tissues compared to normal ovarian tissue. Moreover, FOXP4 expression is higher in patients with late-stage OC than in those with early-stage OC. Inhibition of FOXP4 expression significantly suppresses cell proliferation, although it has little effect on apoptosis. Additionally, FOXP4 knockout markedly reduces the invasive and metastatic potential of OC cells (Ji et al., 2024).

The expression of FOXP4 appears to be regulated by the Wnt/β-catenin signaling pathway. Activation of this pathway using Wnt agonists or β-catenin overexpression upregulates FOXP4 expression in OC cells. FOXP4 itself acts as a positive regulator of Wnt/β-catenin signaling. Silencing FOXP4 leads to reduced expression of key downstream targets, including TCF/LEF1, CCND1, GSK3B, and c-Myc. The mechanism by which FOXP4 enhances Wnt/β-catenin pathway activity may involve PTK7 (Ji et al., 2024).

FOXP3 serves as a definitive marker of Tregs, which play a major immunosuppressive role in the tumor microenvironment of OC. Tregs inhibit the activation and proliferation of immune effector cells such as CD4^+^ and CD8^+^ T cells through the secretion of immunosuppressive cytokines, including interleukin-10 and transforming growth factor-beta. This suppressive activity facilitates immune evasion by tumor cells, allowing OC to progress and proliferate within the host (Shi et al., 2024).

## FOXQ

FOXQ1 is upregulated in various malignancies, including hepatocellular carcinoma, non–small-cell lung cancer, colorectal cancer, and breast cancer. It plays a pivotal role in regulating EMT, contributing to tumor invasion, metastasis, and poor prognosis. FOXQ1 is not expressed in normal ovarian epithelium, whereas its expression is significantly elevated in OC tissues (Xiang et al., 2020; Luo et al., 2021). High FOXQ1 expression is associated with reduced overall survival and progression-free survival. In a study involving 10 OC cell lines, FOXQ1 was found to be highly expressed in TOV-21G and OVCA-429 cells, while its expression was relatively low in OVCA-R3 and TOV112D cells. Both *in vitro* and *in vivo* experiments demonstrated that FOXQ1 knockdown significantly impairs the invasive and metastatic capabilities of OC cells (Wu et al., 2024).

Aberrant FOXQ1 expression influences several key signaling pathways, including the Wnt, MAPK, and Hippo pathways. Among these, the Wnt signaling pathway appears to be a primary mediator of FOXQ1-induced invasion and metastasis in OC. Modulation of FOXQ1 expression directly affects levels of β-catenin, c-Myc, and cyclin D1, aligning with the downstream targets of other FOX family members implicated in ovarian tumorigenesis. Notably, FOXQ1-driven activation of the Wnt pathway is linked to LAMB3, a key component in this signaling cascade.

The regulatory mechanisms of FOXQ1 expression involve both transcriptional and post-transcriptional controls. Nucleus accumbens-associated protein 1 (NAC1), a member of the bric-a-brac-tramtrack-broad protein family, forms higher-order transcriptional complexes by interacting with DNA-binding cofactors. In OC, NAC1 overexpression is associated with increased invasiveness, chemoresistance, and tumor recurrence. NAC1 enhances cancer cell migration, promotes autophagy via the high-mobility group box 1 pathway under cisplatin exposure, supports cell survival, and suppresses senescence. NAC1 and FOXQ1 are co-expressed in high-grade serous OC, and FOXQ1 knockdown significantly diminishes the oncogenic effects driven by NAC1 (Gao et al., 2014). Furthermore, Gao et al. reported that BCL6 induces FOXQ1 transcription in OC cells via a mechanism that depends on the transcriptional cofactor NAC1. Several BCL6-binding sites have been identified in the FOXQ1 promoter, with at least one being essential for FOXQ1 activation (Gao et al., 2020). NAC1 is both necessary and sufficient for maintaining FOXQ1 expression (Gao et al., 2014), suggesting that disruption of NAC1 dimerization offers a therapeutic strategy to downregulate FOXQ1.

FOXQ1 promotes OC progression through the LAMB3/Wnt/β-catenin signaling axis. Poly (ADP-ribose) polymerase 1 (PARP1) stabilizes FOXQ1 by inhibiting its proteasomal degradation via suppression of the E3 ubiquitin ligase HSC70-interacting protein (CHIP). *In vivo* combination therapy studies and clinical prognostic analyses have shown that PARP1 facilitates OC progression by stabilizing FOXQ1 and activating the LAMB3/Wnt/β-catenin pathway (Luo et al., 2021). The PARP inhibitor niraparib significantly suppressed tumor growth in a mouse xenograft model of FOXQ1-expressing OC, suggesting that PARP inhibition may offer therapeutic benefit by targeting FOXQ1 (Wu et al., 2024). Mechanistically, FOXQ1 is a substrate of CHIP, and PARP1 disrupts the FOXQ1-CHIP interaction, thereby preventing proteasomal degradation and increasing FOXQ1 protein levels (Wu et al., 2024). In addition to PARP1 inhibitors, other molecules such as ubiquitin-specific peptidases, which regulate protein degradation, may influence FOXQ1 expression. Although some ubiquitin-specific peptidases promote FOXQ1 stability and expression (Wang J. et al., 2024), further validation of their role in OC is warranted (Figure 1).

## FOXR

The FOXR subgroup consists of FOXR1 and FOXR2, which share 57.7% genetic similarity. FOXR1 is expressed in multiple organs, including the nervous and reproductive systems (Cheung et al., 2018). FOXR1 expression in ovarian granulosa cells is significantly elevated in women aged 30–39 years (Liu et al., 2023). The FOXR1 fusion gene has been implicated as an oncogene in various malignancies, including neuroblastoma (Katoh et al., 2013) and lymphoma (Pommerenke et al., 2016). FOXR2, located on the X chromosome at Xp11.21, is considered a testis-specific gene, as it is normally expressed only in the testes. However, FOXR2 is also recognized as an oncogenic factor that, when mutated or overexpressed, can contribute to tumorigenesis (Laissue, 2019).

FOXR2 is frequently upregulated in OC, with this overexpression correlating with poorer histological grades and reduced survival rates (Li et al., 2018). Elevated FOXR2 expression is associated with increased cell proliferation, migration, EMT, and drug resistance. Silencing FOXR2 suppresses these malignant phenotypes. Moreover, FOXR2 overexpression promotes angiogenesis by upregulating vascular endothelial growth factor. This oncogenic effect may be mediated through the activation of the Hedgehog signaling pathway, which is known to drive angiogenesis, metastasis, and progression in OC. Inhibition of this pathway using sonidegib reduces FOXR2-induced cell migration and lung metastasis. Interestingly, the Hedgehog pathway also regulates FOXR2 activity, suggesting a reciprocal regulatory relationship in which FOXR2 functions as both an upstream modulator and downstream effector of Hedgehog signaling (Li et al., 2018). Additionally, FOXR2 parallels the oncogenic activity of MYC. FOXR2 can directly bind to the promoter region of the MYC gene, enhancing its transcriptional activity. As a result, increased FOXR2 expression is often accompanied by elevated MYC levels (Li et al., 2016; schmitt-Hoffner et al., 2021). Given this relationship, the combined use of FOXR2 inhibitors and BET inhibitors such as JQ1—which suppress MYC transcription—may offer enhanced therapeutic efficacy. However, direct evidence supporting this combination strategy in OC is currently lacking (Figure 1) Small regulatory RNAs also modulate FOXR2 expression in OC.

Zhang et al. identified FOXR2 as a novel target of miR-1252 (Zhang S. et al., 2020). In PTX-resistant OC cells, the circular RNA circCELSR1 was found to upregulate FOXR2 by sponging miR-1252. Furthermore, another circular RNA, circANKRD17 (also known as circ0007883), stabilizes FOXR2 by binding to FUS, an RNA-binding protein. CircCELSR1 is significantly overexpressed in OC samples and is associated with PTX resistance (Liang et al., 2023). Silencing circCELSR1 enhances the cytotoxic effect of PTX in OC cells, induces G0/G1 cell cycle arrest, and increases apoptosis.

## Discussion

The FOX protein family comprises numerous members, and current strategies for targeting FOX proteins primarily focus on transcriptional regulation, translation, post-translational modifications, and downstream signaling pathways. Although the mechanisms governing transcription, translation, and post-translational modifications are partially shared across FOX family members, they also exhibit notable differences. For example, phosphorylation, acetylation, ubiquitination, and proteasomal degradation can modulate the expression and activity of various FOX proteins. Therefore, manipulating post-translational modifications may upregulate specific FOX proteins, but it could also inadvertently alter the expression of other members, given the overlapping regulatory mechanisms. The functional roles of FOX proteins in tumors are diverse and context-dependent. In OC, members such as FOXA1, FOXM1, FOXP4, FOXQ1, and FOXR2 primarily exhibit oncogenic functions, whereas others like FOXO and FOXP1 are associated with tumor-suppressive effects. Combination therapies targeting downstream effectors of FOX proteins represent a promising approach; however, the high degree of heterogeneity in OC complicates the identification of consistent downstream targets across different patients. This heterogeneity may be a key factor contributing to the limited clinical success of current FOX-targeted therapies. Given the large number of FOX family members, existing research has largely focused on elucidating the role of individual FOX proteins in OC, resulting in a lack of comprehensive insight into the broader functional landscape of the entire family. Future studies should aim to evaluate the systemic effects of pharmacological interventions on multiple FOX proteins, with particular emphasis on post-translational regulatory mechanisms. Additionally, further investigation is needed to identify and develop more specific therapeutic agents that account for subgroup-specific differences among FOX proteins.

## Prospects

Despite significant advances in FOX-targeted therapies for OC, several challenges remain: 1. Overlapping regulatory mechanisms and functional divergence: Although many FOX proteins share regulatory pathways, such as phosphorylation and deacetylation, their functional outcomes can vary markedly. For instance, phosphorylation of FOXO by AKT inhibits its nuclear translocation and reduces its transcriptional activity, thereby diminishing its tumor-suppressive function. In contrast, phosphorylation of FOXM1 by AKT promotes its nuclear localization and enhances its oncogenic activity. Thus, the PI3K/AKT signaling pathway accelerates OC progression by simultaneously suppressing FOXO and activating FOXM1. However, it remains unclear whether this pathway similarly influences other FOX family members, highlighting a significant gap in our understanding. Comprehensive investigations into how various signaling pathways and treatments affect all FOX proteins are essential. 2. Limited clinical application of gene editing: Gene editing technologies such as CRISPR/Cas9 have been widely employed in basic research. For example, a knockout FOXR1 zebrafish model has been established to study its biological function (Cheung et al., 2018), and CRISPR/Cas9 has been used to investigate the binding properties of FOXM1 to gene promoters (Chen et al., 2025). However, the clinical translation of such gene-editing approaches remains limited. 3. Challenges in drug design and specificity: While computer-aided drug design has yielded promising results in developing FOX-targeted therapies (Zhou Z. Y. et al., 2022), few related clinical studies have been conducted to date. Moreover, the structural similarity among FOX family members presents a significant obstacle in identifying highly specific inhibitors. Future efforts should focus on designing more selective therapeutic agents to minimize off-target effects and improve treatment efficacy.
